# Test–retest reliability and minimal detectable change of corticospinal tract integrity in chronic stroke

**DOI:** 10.1002/hbm.24961

**Published:** 2020-02-24

**Authors:** Allison F. Lewis, Makenzie Myers, Jenny Heiser, Melissa Kolar, Jessica F. Baird, Jill C. Stewart

**Affiliations:** ^1^ Department of Exercise Science University of South Carolina Columbia South Carolina

**Keywords:** corticospinal tract, diffusion tensor imaging, fractional anisotropy, minimal detectable change, reliability, stroke

## Abstract

Diffusion tensor imaging (DTI) can be used to index white matter integrity of the corticospinal tract (CST) after stroke; however, the psychometric properties of DTI‐based measures of white matter integrity are unknown. The purpose of this study was to examine test–retest reliability as determined by intraclass correlation coefficients (ICC) and calculate minimal detectable change (MDC) of DTI‐based measures of CST integrity using three different approaches: a Cerebral Peduncle approach, a Probabilistic Tract approach, and a Tract Template approach. Eighteen participants with chronic stroke underwent DTI on the same magnetic resonance imaging scanner 4 days apart. For the Cerebral Peduncle approach, a researcher hand drew masks at the cerebral peduncle. For the Probabilistic Tract approach, tractography was seeded in motor areas of the cortex to the cerebral peduncle. For the Tract Template approach, a standard CST template was transformed into native space. For all approaches, the researcher performing analyses was blind to participant number and day of data collection. All three approaches had good to excellent test–retest reliability for fractional anisotropy (FA; ICCs >0.786). Mean diffusivity, axial diffusivity, and radial diffusivity were less reliable than FA. The ICC values were highest and MDC values were the smallest for the most automated approach (Tract Template), followed by the combined manual/automated approach (Probabilistic Tract) then the manual approach (Cerebral Peduncle). The results of this study may have implications for how DTI‐based measures of CST integrity are used to define impairment, predict outcomes, and interpret change after stroke.

## 1 INTRODUCTION

The corticospinal tract (CST) is an important descending white matter pathway for the control of skilled movement. The CST receives fibers from the primary motor cortex (M1), as well as from sensory and secondary motor areas including somatosensory cortex (S1), dorsal premotor cortex (PMd), ventral premotor cortex (PMv), supplementary motor area (SMA), and presupplementary motor area (preSMA; Archer, Vaillancourt, & Coombes, 2017; Dum & Strick, 1991; Nudo, 2007). The integrity of the CST is often compromised after stroke resulting in motor impairment that persists into the chronic phase of stroke. Microstructural changes in the CST due to direct lesion or axonal degradation related to remote lesion after stroke can be detected and measured by diffusion‐weighted magnetic resonance imaging (MRI; Arfanakis et al., 2002; Thomalla et al., 2004).

Diffusion tensor imaging (DTI) can be used to quantify the microstructural integrity of white matter tracts (Le Bihan & Johansen‐Berg, 2012; O'Donnell & Westin, 2011). CST integrity, as measured by DTI, has been shown to correlate with level of motor impairment (Burke et al., 2014; Lindenberg et al., 2010; Stinear et al., 2007), predict long term motor outcomes (Groisser, Copen, Singhal, Hirai, & Schaechter, 2014; Puig et al., 2013), and predict treatment gains after stroke (Byblow, Stinear, Barber, Petoe, & Ackerley, 2015; Lindenberg, Zhu, Rüber, & Schlaug, 2012; Stinear et al., 2007). Taken together, CST integrity has been suggested as a prognostic biomarker for motor system recovery after stroke (Boyd et al., 2017; Puig et al., 2017). Recently, studies have examined whether DTI‐based measures of white matter integrity change over time or in response to training (Caeyenberghs et al., 2018; Schlaug, Marchina, & Norton, 2009; Wan, Zheng, Marchina, Norton, & Schlaug, 2014; Zheng & Schlaug, 2015). However, the psychometric properties of DTI‐based measures of white matter integrity, like reliability and estimated measurement noise, have not been fully described.

Diffusion imaging‐based measures of white matter integrity are reproducible and reliable between observers, scanners, and across time in nondisabled populations (Albi et al., 2017; Danielian, Iwata, Thomasson, & Floeter, 2010; Fox et al., 2012; Heiervang, Behrens, Mackay, Robson, & Johansen‐Berg, 2006; Kristo et al., 2013; Lin et al., 2013; Wakana et al., 2007). Fewer studies have investigated the reliability of diffusion imaging‐based white matter integrity after stroke (Borich, Wadden, & Boyd, 2012; Lin et al., 2013; Snow et al., 2016). In individuals with chronic stroke, inter‐ and intra‐rater reliability of diffusion imaging‐based measures of CST integrity has been shown to be reliable (Borich et al., 2012; Lin et al., 2013). Yet, only a single study has examined test–retest reliability by calculating intraclass correlation coefficients (ICC) in chronic stroke (Snow et al., 2016). The results suggested that test–retest reliability of measures of CST integrity in individuals with chronic stroke is excellent, however, study limitations (relatively small sample size, variance in the number of days between test and retest scans, investigation of a single tractography approach) may impact the application of these findings.

Minimal detectable change (MDC) values represent the smallest amount of change required to exceed the inherent variability of a measure. These values are necessary for determining whether clinical interventions result in change beyond estimated measurement noise (Portney & Watkins, 2008). To the best of our knowledge, no previous study has reported MDC values of DTI‐based measures of CST integrity after stroke. Since a gold standard approach for measuring CST integrity after stroke has not been established and ICC and MDC values are unique to the approach utilized, investigation of the psychometric properties of several different approaches for determination of CST integrity is needed. Common approaches for integrity measurement include single region ROI masks (manually drawn or from an atlas), full tract templates transformed from standard space to native space, and probabilistic tractography with ROIs (manually drawn or from an atlas) to isolate the tract of interest.

While many pathways may contribute to the movement (Fling & Seidler, 2012; Rodríguez‐Herreros et al., 2015; Stewart et al., 2017), the CST is the only white matter pathway that has been recommended by expert consensus as a biomarker of the motor system after stroke. Therefore, the purpose of this study was to determine the test–retest reliability and to estimate MDC of DTI‐based measures of CST integrity in chronic stroke. We hypothesized that DTI‐based measures of CST integrity would have good to excellent test–retest reliability, with more automated approaches (Tract Template and Probabilistic Tractography approach) having higher reliability and smaller MDCs than manual, region of interest (ROI) approaches (Cerebral Peduncle approach).

## 2 MATERIALS AND METHODS

### 2.1 Participants

Structural images, diffusion‐weighted images, and behavioral assessments were obtained from 18 individuals in the chronic phase of stroke 4 days apart as part of a larger study (http://clinicaltrials.gov Identifier: NCT02785419) that examined brain activity in response to a brief period of practice after stroke. Practice occurred on 4 consecutive days and consisted of movement of a joystick with the more‐impaired hand based on a visual cue for 30–45 min (168–240 movement repetitions per day) on all 4 days. This amount of behavioral practice is well below the intensity hypothesized to produce structural brain changes (Scholz, Klein, Behrens, & Johansen‐Berg, 2009). Per protocol of the larger study, individuals were eligible to participate if they were ≥18 years old, in the chronic phase of stroke recovery (>6 months poststroke), right hand dominant (Oldfield, 1971), scored ≥19 on the Montreal Cognitive Assessment (Nasreddine et al., 2005), showed evidence of upper extremity impairment by an upper extremity Fugl‐Meyer (UE FM) score < 66 (Fugl‐Meyer, Jääskö, Leyman, & Olsson, 1975) and/or at least 15% deficit on the Nine Hole Peg Test (Grice et al., 2003) on the more impaired hand compared to the less impaired hand and demonstrated some movement ability as shown by an UE FM score >30 and/or the ability to move at least one block on the Box and Blocks Test with the affected upper extremity (Mathiowetz, Federman, & Wiemer, 1985). Individuals were excluded if they had any acute medical problems, severe ideomotor apraxia as defined by a score ≤65 on the Test of Upper Limb Apraxia (Vanbellingen et al., 2010), hemispatial neglect with <52 on the BIT Star Cancelation Test (Hartman‐Maeir & Katz, 1995), significant arm pain that interfered with movement, contraindications to MRI scanning (e.g., metal implants, claustrophobia), or a history of other, nonstroke related neurological disorder. All participants provided written consent, and all aspects of this study were approved by the University of South Carolina Institutional Review Board.

### 2.2 Image acquisition

All images were acquired on a Siemens Prisma 3 Tesla MRI scanner with a 20‐channel head coil at the University of South Carolina's McCausland Center for Brain Imaging. High resolution T1‐weighted structural images (TR = 2,250 ms, TE = 4.11 ms, 192 sagittal slices, 1 mm^3^ isotropic voxels) and T2‐weighted structural images (T2 = 3,200 ms, TE = 567 ms, 176 slices, 1 mm^3^ isotropic voxels) were acquired on Day 1. Diffusion‐weighted images were collected using an echo‐planar imaging sequence (TR = 3,839 ms, TE = 71 ms, 68 slices, 1.8 mm^3^ isotropic voxels, 56 noncollinear directions, *b* = 1,000 s/mm^2^). Whole‐brain diffusion‐weighted images (DWI) were acquired on Day 1 and again 4 days later (Day 4). Two runs of diffusion images were acquired on each day with reverse encoding directions (anterior to posterior and posterior to anterior); seven b0 volumes were acquired in each run.

### 2.3 Image preprocessing

#### 2.3.1 Diffusion image preprocessing

All DWI image processing was completed in FMRIB's Software Library (FSL; FMRIB Center, Oxford, UK) using the FMRIB's Diffusion Toolbox (FDT). Volumes without diffusion weighting (b0 volumes) were extracted from both phase‐encoding directions, merged, and utilized to estimate susceptibility induced distortions using FSL's top‐up command (Andersson, Skare, & Ashburner, 2003; Smith et al., 2004). The skull and dura were removed (Smith, 2002) and images were corrected for the eddy current‐induced off‐resonance fields (Andersson & Sotiropoulos, 2016). Voxelwise maps of fractional anisotropy (FA), mean diffusivity (MD), three eigenvectors, and three eigenvalues were created using DTIFIT (Behrens et al., 2003). Images were visually inspected for quality during each step of preprocessing. Bedposting was completed to build distributions of diffusion parameters at each voxel (Behrens, Berg, Jbabdi, Rushworth, & Woolrich, 2007).

#### 2.3.2 Structural image preprocessing

FSL's Brain Extraction Tool was used to perform brain extraction using robust brain center estimation and thresholding to maintain the inclusion of lesioned and exclude extraneous nonbrain tissues (Smith, 2002). A trained researcher hand drew stroke lesion masks on the T2 structural image. All lesion masks were checked by a second, experienced researcher. The T2 lesion mask was linearly registered to the structural T1, then binarized to be used as a weighting volume during registration. The lesion mask volume was deweighted during all linear and nonlinear registration processes (Schulz et al., 2017a). The structural T1 image was linearly then nonlinearly registered into diffusion space using FSL's FLIRT and FNIRT (Jenkinson & Smith, 2001; Smith et al., 2004).

### 2.4 Data processing

FA, MD, radial diffusivity (RD), and axial diffusivity (AD) were extracted from the diffusion data. Directional diffusivities were determined by three eigenvalues, where AD is equal to the first eigenvalue (AD = λ_1_) and RD is equal to the average of the second and third eigenvalue (RD = λ_2_ + λ_3_/2; Basser, 1995). FA is a ratio value derived from the eigenvalues that represents the directional preference of water diffusion within the structural bounds of tissue.

Three approaches were used to extract FA, MD, AD, and RD from the ipsilesional and contralesional CST in each participant's native space: Cerebral Peduncle, Probabilistic Tract, and Tract Template. In all approaches, the masks used for data extraction were thresholded to voxels with an FA > 0.2. Separate researchers completed the data analysis for each approach; all researchers were blinded to the subject number and day of data collection during analyses. The researchers (A.L., M.M., J.H.) were trained in their respective approach by an experienced researcher (J.S.). The final cerebral peduncle masks, probabilistic tracts, and tract transformations were all visually inspected by A.L. or J.S.

FA ratio and FA asymmetry represent lesioned tract integrity normalized to the tract of the nonlesioned hemisphere. FA ratio values were calculated by dividing the ipsilesional tract FA value by the contralesional tract value, where a value of 1 indicates symmetrical tract integrity (FA_lesion_/FA_nonlesion_). FA asymmetry was calculated by taking the difference of the two tracts and dividing by the sum of the two tracts, where a value of zero indicates symmetrical tract integrity ((FA_nonlesion_ – FA_lesion_)/(FA_nonlesion_ + FA_lesion_); Stinear et al., 2007).

#### 2.4.1 Cerebral peduncle approach

**Figure 1:**
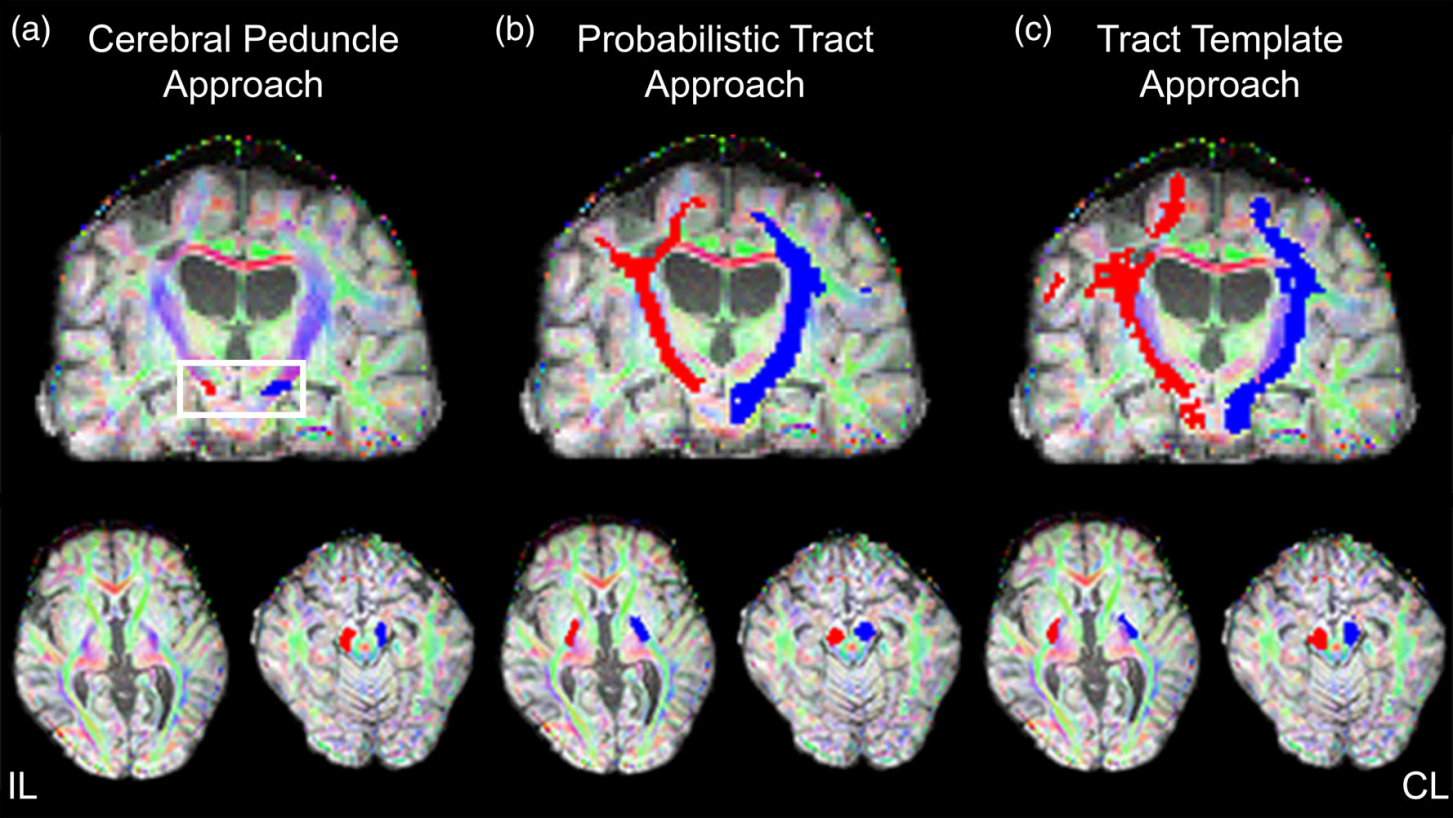
Visualization of each approach for a single participant, where red is the ipsilesional (IL) tract and blue is the contralesional (CL) tract. (a) Cerebral Peduncle Approach with resulting thresholded cerebral peduncle ROI mask. The white box highlights the region where the ROI mask was drawn. (b) Probabilistic Tract Approach with resulting normalized, thresholded, and binarized 3D mask of probabilistic tract for the CST. (c) Tract Template Approach with resulting thresholded 3D tract mask from template transformation

A single researcher hand drew masks on the three contiguous axial slices that showed the largest cross‐sectional area of the cerebral peduncle (Mark et al., 2008; Schaechter, Perdue, & Wang, 2008). The T1 structural image was registered to native diffusion space. Next, the colored FA map was overlaid on the T1 structural image in native diffusion space for ROI mask drawing (Figure 1a). All masks were checked by a second researcher. The stroke lesion did not overlap with the cerebral peduncle ROI in any participant. The peduncle masks were thresholded, where voxels with FA < 0.2 were removed. The thresholded masks were used to extract mean FA, MD, AD, and RD from each participant's native space.

#### 2.4.2 Probabilistic tract approach

Standard human motor area templates (HMATs; Mayka, Corcos, Leurgans, & Vaillancourt, 2006) of M1, PMd, PMv, SMA, preSMA, and S1 were registered to the individual participant's diffusion space, with stroke lesions weighted to zero during the registration process using FSL's FLIRT and FNIRT (Jenkinson & Smith, 2001; Smith et al., 2004). The HMATs were used to seed probabilistic tractography, creating an individual descending probable tract from each HMAT in each hemisphere.

Tractography was completed using the PROBTRACKX2 command in FSL (maximum number of steps = 2000, step length = 0.5 mm, number of samples = 5,000, curvature thresholds = 0.2, volume fraction before subsidiary fiber volume threshold = .01) with the HMAT as the seed region and waypoints in the PLIC and the cerebral peduncle (waycondition “AND”). Three exclusion masks were drawn to limit extraneous fibers that crossed midline, extended into the cerebellum, or were likely part of the alternate motor pathway in the tegmentum pontis. Each participant had six tracts per hemisphere, one for each of the HMATs.

Each tract was normalized by dividing the distribution by the waytotal and thresholding to include only voxels with at least 1% of total successful streamlines (Schulz et al., 2017b). The six‐component descending tracts for each hemisphere were combined to create a final CST tract for the ipsilesional and contralesional hemisphere. The final tract was thresholded at an FA value of 0.2 (Figure 1b) and used to extract FA, MD, AD, and RD. All registration processes and probabilistic tractography were performed separately for Day 1 and Day 4 data.

#### 2.4.3 Tract template approach

A standard Sensorimotor Area Tract Template (SMATT; Archer, Vaillancourt, et al., 2017) was transformed from standard MNI space into each participant's native diffusion space using linear (FLIRT) and nonlinear (FNIRT) registrations, with the stroke lesion mask weighted to zero. First, a linear registration from T1 to MNI space was created using the brain extracted T1 image, the MNI152 1 mm standard brain template and an inverse binarized T1 stroke lesion mask. Next, the linear transform was used to create the nonlinear warp from T1 to MNI space. The nonlinear warp was then inversed and applied to the SMATT masks to nonlinearly transform the SMATTs from MNI space to native T1 space. Next, a linear transform from T1 to diffusion space was used to create a nonlinear warp using the T1 image, the FA image, and an inverse binarized T1 stroke mask. The T1 to FA nonlinear warp was then applied to the SMATTs in T1 space, resulting in a right and left SMATT in diffusion space. This process was repeated separately for Day 1 and Day 4 data for each participant. The Tract Templates (Figure 1c), registered to the participant's diffusion space, were thresholded at an FA value of 0.2 and used to extract mean FA, MD, RD, and AD.

### 2.5 Statistical analysis

Means for each diffusion measure (FA, FA ratio, FA asymmetry, MD, RD, and AD) were calculated for ipsilesional and contralesional CST, for Day 1 and Day 4, for all three approaches. Differences between mean values of contralesional and ipsilesional CST were evaluated using paired *t* tests. To assess test–retest reliability, intraclass correlation coefficients (ICC) were calculated for two‐way mixed effects, single measurement, with absolute agreement. ICC estimates were interpreted based on the following guidelines: <0.5 indicates poor reliability, 0.5–0.75 indicates moderate reliability, 0.75–0.9 indicates good reliability, and >0.9 indicates excellent reliability (Koo & Li, 2016). ICC values were interpreted considering the 95% confidence interval. Minimal detectable change values were calculated for the 95% confidence interval (MDC = *z*‐score * SEM * √2, where *SEM* (Standard Error Measure) = standard deviation of Day 1 measurement * √(1 − ICC); Portney & Watkins, 2008). Minimal detectable change represents the least amount of change required to exceed the noise or inherent variability of the measurement. Finally, the ipsilesional CST FA, FA ratio, and FA asymmetry were correlated with a measure of upper extremity impairment (UE FM); a *p*‐value corrected for the number of approaches (*p* < .0167) was used to determine significance.

## 3 RESULTS

### 3.1 Participants

**Figure 2:**
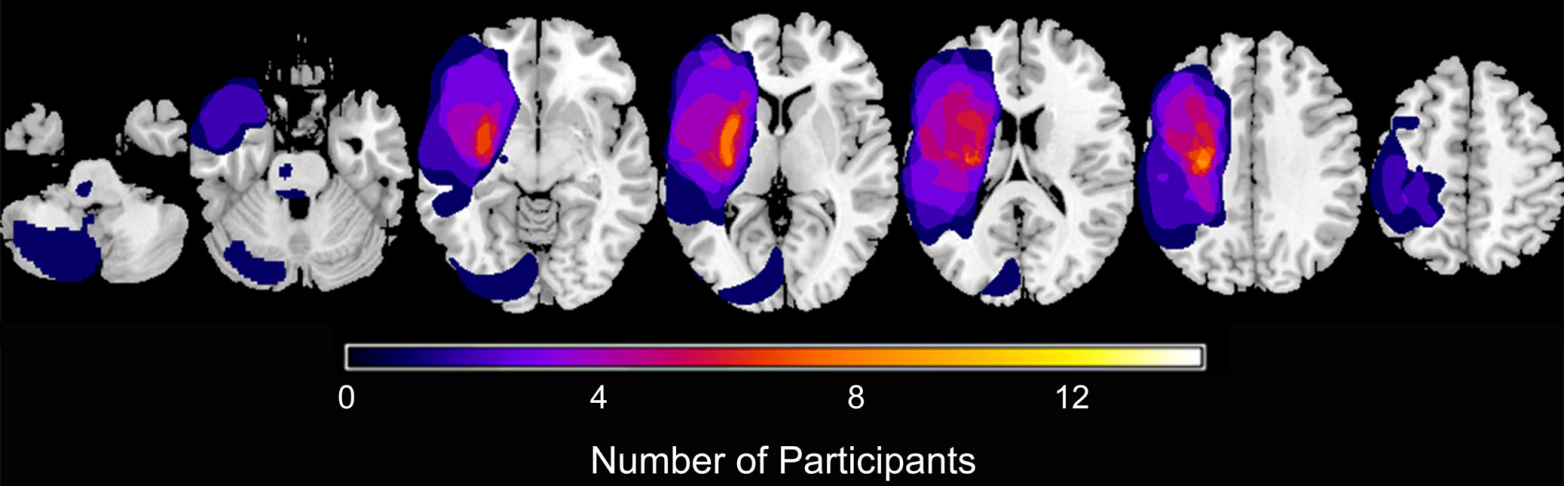
Summary mask of stroke lesions created in MRIcron (http://www.mccauslandcenter.sc.edu/micro/mricron/). Color reflects number of individuals with a stroke in that region. Lesions in the right hemisphere were flipped to the left hemisphere

Participants were on average 57.9 ± 10.4 years old, 41.8 ± 41.7 months post‐stroke, and presented with mild to moderate upper extremity impairment (UE FM 43.1 ± 14.5; Box and Blocks 26.2 ± 16.2). Lesion side, location, and size varied (Table 1, Figure 2). Fifteen out of the 18 participants had a stroke lesion that overlapped with the Tract Template suggesting that most participants had some degree of direct damage to the CST. Despite this, we were able to successfully generate a descending CST in the lesioned hemisphere in all participants (Figure S1).

**Table 1:** Individual participant demographics

Subject	Sex	Age (years)	Lesion side	Lesion location	Lesion volume (mm^3^)	Time post stroke (months)	UE FM	Box and blocks ratio
102	M	54	L	C/SC	187,373	64	54	37/48
104	M	67	L	C/SC	113,222	158	51	50/51
105	F	63	L	SC	3,047	49	47	22/34
108	M	65	L	C/SC	67,729	113	47	33/49
111	M	61	L	BS	188	41	26	13/42
114	M	40	R	CB/BS	23,017	53	61	48/51
116	M	56	R	SC	14,993	13	24	8/53
117	M	47	R	SC	3,347	11	21	4/68
118	M	61	L	BS	199	12	52	44/55
120	M	57	L	SC	1,635	6	36	28/46
121	F	53	R	C/SC	30,003	13	43	23/31
124	F	67	R	SC	6,170	22	28	2/43
125	F	60	R	SC	3,691	7	59	44/48
126	F	35	L	BS	810	41	59	16/51
128	F	64	R	C/SC	16,299	12	54	36/44
133	M	56	L	SC	1,044	22	18	2/45
135	M	69	L	C/SC	105,620	35	47	21/55
136	M	76	L	C/SC	30,599	21	57	41/45

Abbreviations: Box and Blocks ratio, number of blocks moved with affected hand/number of blocks moved with unaffected hand; BS, brainstem; C, cortical; CB, cerebellum; SC, subcortical; UE FM, Upper Extremity Fugl‐Meyer, with maximum score of 66 meaning less impairment.

### 3.2 Corticospinal tract integrity

FA values were significantly lower for the ipsilesional tract as compared to the contralesional tract for the Cerebral Peduncle, Probabilistic Tract, and Tract Template approaches (*p* < .01; Table 2). Mean FA ratio was relatively high (>0.90) and mean FA asymmetry was relatively low (<0.06) across approaches and days. MD and RD values were significantly higher for the ipsilesional CST compared to the contralesional CST (*p* < .01) for all three approaches. AD values were significantly higher for the ipsilesional CST compared to the contralesional CST (*p* < .05) for all three approaches, except for Cerebral Peduncle approach measured on Day 4 (*p* = .066).

**Table 2:** Tract variables, ICC, and MDC_95_ by approach

	Mean Day 1 [Mean values (SD)]	Mean Day 4 [Mean values (SD)]	ICC	ICC CI	ICC rating	MDC_95_
*Cerebral peduncle*						
FA						
Ipsilesion	0.569^a^ (0.052)	0.566^a^ (0.062)	0.844^b^	0.630–0.939	Good	0.057
Contralesion	0.624 (0.044)	0.628 (0.046)	0.786^b^	0.513–0.914	Good	0.056
Ratio	0.912 (0.074)	0.903 (0.076)	0.828^b^	0.604–0.932	Good	0.085
Asymmetry	0.048 (0.041)	0.053 (0.042)	0.833^b^	0.614–0.934	Good	0.046
MD (×10^−3^ mm^2^/s)						
Ipsilesion	1.048^a^ (0.092)	1.041^a^ (0.077)	0.493^c^	0.035–0.776	Poor	0.182
Contralesion	0.942 (0.074)	0.941 (0.077)	0.650^c^	0.267–0.854	Moderate	0.121
AD (×10^−3^ mm^2^/s)						
Ipsilesion	1.760^a^ (0.152)	1.749 (0.125)	0.764^b^	0.472–0.905	Good	0.205
Contralesion	1.674 (0.088)	1.676 (0.075)	0.740^b^	0.423–0.895	Moderate	0.125
RD (×10^−3^ mm^2^/s)						
Ipsilesion	0.693^a^ (0.085)	0.688^a^ (0.089)	0.526^c^	0.079–0.794	Moderate	0.162
Contralesion	0.576 (0.084)	0.574 (0.092)	0.707^b^	0.363–0.880	Moderate	0.126
*Probabilistic tract*						
FA						
Ipsilesion	0.461^a^ (0.033)	0.459^a^ (0.032)	0.973^b^	0.930–0.990	Excellent	0.015
Contralesion	0.490 (0.021)	0.489 (0.019)	0.970^b^	0.922–0.988	Excellent	0.010
Ratio	0.939 (0.043)	0.938 (0.040)	0.936^b^	0.837–0.975	Excellent	0.030
Asymmetry	0.032 (0.023)	0.032 (0.021)	0.932^b^	0.828–0.974	Excellent	0.017
MD (×10^−3^ mm^2^/s)						
Ipsilesion	0.842^a^ (0.072)	0.844^a^ (0.071)	0.986^b^	0.965–0.995	Excellent	0.023
Contralesion	0.767 (0.022)	0.767 (0.023)	0.951^b^	0.873–0.981	Excellent	0.014
AD (×10^−3^ mm^2^/s)						
Ipsilesion	1.297^a^ (0.098)	1.299^a^ (0.097)	0.993^b^	0.982–0.997	Excellent	0.023
Contralesion	1.222 (0.036)	1.221 (0.031)	0.944^b^	0.856–0.979	Excellent	0.024
RD (×10^−3^ mm^2^/s)						
Ipsilesion	0.614^a^ (0.066)	0.617^a^ (0.066)	0.980^b^	0.948–0.992	Excellent	0.026
Contralesion	0.539 (0.024)	0.540 (0.025)	0.965^b^	0.910–0.987	Excellent	0.013
*Tract template*						
FA						
Ipsilesion	0.422^a^ (0.025)	0.422^a^ (0.025)	0.984^b^	0.958–0.994	Excellent	0.009
Contralesion	0.455 (0.020)	0.453 (0.020)	0.976^b^	0.930–0.992	Excellent	0.009
Ratio	0.928 (0.040)	0.932 (0.038)	0.977^b^	0.927–0.992	Excellent	0.017
Asymmetry	0.038 (0.022)	0.036 (0.020)	0.975^b^	0.923–0.991	Excellent	0.009
MD (×10^−3^ mm^2^/s)						
Ipsilesion	0.837^a^ (0.071)	0.839^a^ (0.068)	0.987^b^	0.966–0.995	Excellent	0.022
Contralesion	0.765 (0.020)	0.770 (0.019)	0.877^b^	0.667–0.954	Good	0.020
AD (×10^−3^ mm^2^/s)						
Ipsilesion	1.243^a^ (0.087)	1.246^a^ (0.086)	0.989^b^	0.971–0.996	Excellent	0.025
Contralesion	1.177 (0.023)	1.182 (0.027)	0.882^b^	0.694–0.956	Good	0.022
RD (×10^−3^ mm^2^/s)						
Ipsilesion	0.634^a^ (0.065)	0.635^a^ (0.063)	0.986^b^	0.963–0.995	Excellent	0.021
Contralesion	0.559 (0.025)	0.564 (0.024)	0.926^b^	0.778–0.974	Excellent	0.019

Abbreviations: AD, axial diffusivity (×10^−3^ mm^2^/s); CI, 95% confidence interval; FA asymmetry = (FA_contralesion_ − FA_ipsilesion_)/(FA_contralesion_ + FA_ipsilesion_); FA ratio = FA_ipsilesion_/FA_contralesion_; FA, fractional anisotropy; ICC, intraclass correlation coefficient; MD, mean diffusivity (×10^−3^ mm^2^/s); MDC_95_, minimal detectable change at the 95% confidence interval; RD, radial diffusivity (×10^−3^ mm^2^/s).

a Significant difference at *p* < .05 between ipsilesional value and contralesional value.

b F‐statistic for the ICC is significant at *p* < .001.

c F‐statistic for the ICC is significant at *p* < .02.

**Figure 3:**
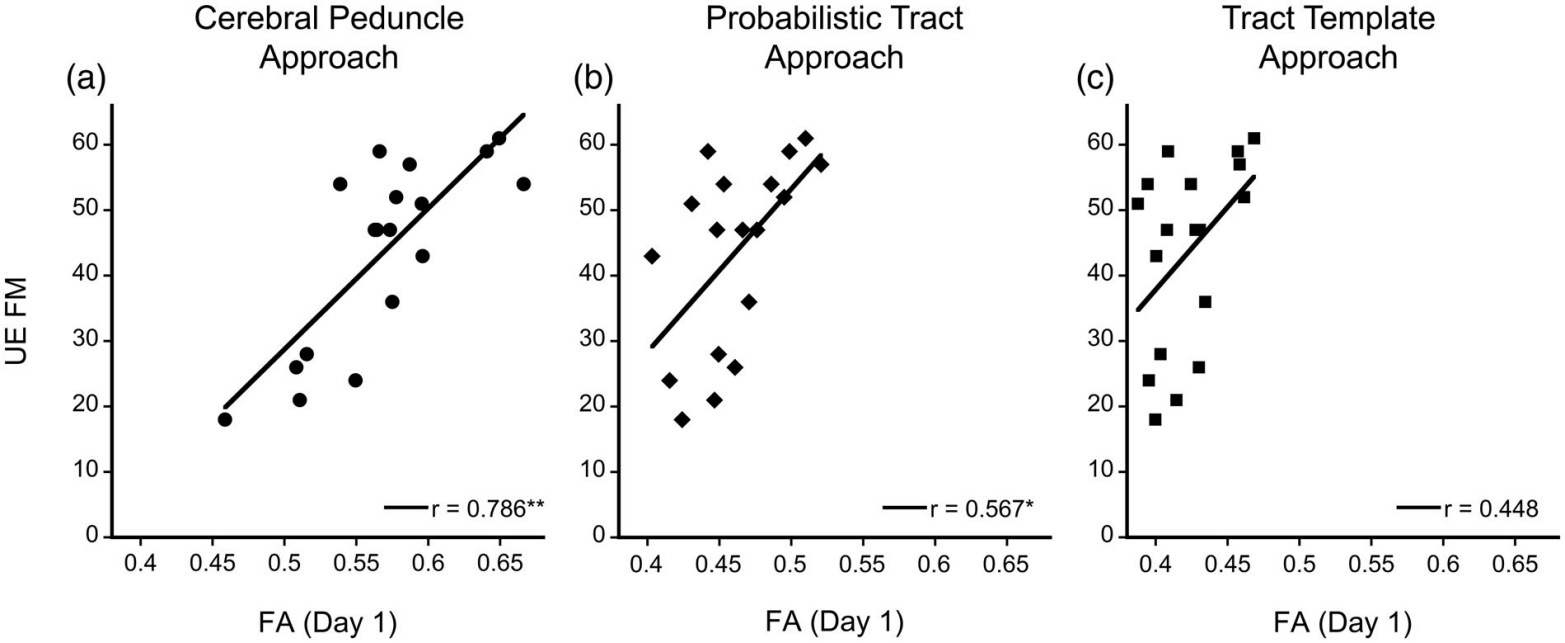
Ipsilesional CST (corticospinal tract) FA values measured from Day 1 correlated with upper extremity impairment measured by UE FM for each approach: (a) Cerebral Peduncle Approach, (b) Probabilistic Tract Approach, and (C) Tract Template Approach. UE FM, upper extremity Fugl‐Meyer; FA, fractional anisotropy measured on Day 1; *r*, Pearson's correlation coefficient. *Significant at *p* < .05, **p < .01

Overall, FA in the ipsilesional CST correlated with level of motor impairment (UE FM motor score; Figure 3). For the Cerebral Peduncle approach, ipsilesional CST FA values from Day 1 (*r* = .786, *p* < .01) and Day 4 (*r* = .798, *p* < .01) were significantly correlated with UE FM. For the Probabilistic Tract approach, ipsilesional CST FA values from Day 1 (*r* = .567, *p* = .014) and Day 4 (*r* = .632, *p* < .01) were significantly correlated with UE FM. For the Tract Template approach, neither Day 1 nor Day 4 FA values were significantly correlated with UE FM (*r* < .470, *p* > .05). For the Cerebral Peduncle approach, FA ratio (Day 1: *r* = .635, *p* < .01; Day 4: *r* = .740, *p* < .01) and FA asymmetry (Day 1: *r* = −.644, *p* < .01; Day 4: *r* = −.754, *p* < .01) correlated with UE FM motor score. FA ratio and FA asymmetry were not correlated with UE FM for the Probabilistic Tract (*r* < .418, *p* > .084) or Tract Template (*r* < .036, *p* > .886) approach.

### 3.3 Test–retest reliability and minimal detectable change of measures of corticospinal tract integrity

**Figure 4:**
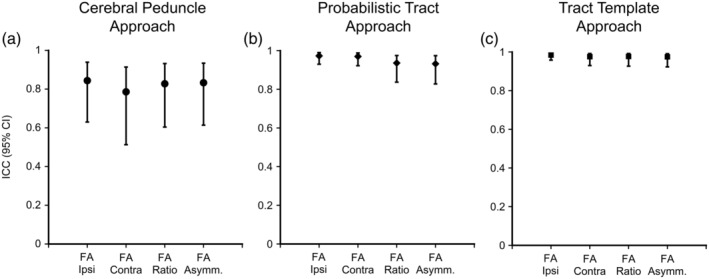
Intraclass correlation coefficients with the 95% confidence interval (ICC [95% CI]) for ipislesional CST FA, contralesional CST FA, FA ratio, and FA asymmetry by approach: (a) Cerebral Peduncle Approach, (b) Probabilistic Tract Approach, and (c) Tract Templeate Approach. CST, corticospinal tract; FA_ipsi_, fractional anisoptropy of the ipslesional CST; FA_contra_, fractional anisotropy of the contralesional CST; FA Ratio, fractional anisotropy ratio (FA_ipsi_/FA_contra_); FA Asymm., fractional anisotropy asymmetry ((FA_contra_ − FA_ipsi_)/(FA_contra_ + FA_ipsi_))

Overall, test–retest reliability for DTI‐derived measures of CST integrity ranged from moderate to excellent (Table 2, Figure 4). All measures of FA (ipsilesional, contralesional, ratio, and asymmetry) had moderate to excellent reliability for the Cerebral Peduncle approach (ICC > 0.786), good to excellent reliability for Probabilistic Tract approach (ICC > 0.932), and excellent reliability for the Tract Template approach (ICC > 0.975). Ipsilesional and contralesional MD had poor to good reliability for the Cerebral Peduncle approach (ICC > 0.493), good to excellent reliability for the Probabilistic Tract approach (ICC > 0.951), and good to excellent reliability for the Tract Template Approach (ICC > 0.877). Ipsilesional and contralesional RD had poor to good reliability for the Cerebral Peduncle approach (ICC > 0.526), excellent reliability for the Probabilistic Tract approach (ICC > 0.965), and good to excellent reliability for the Tract Template Approach (ICC > 0.926). Ipsilesional and contralesional AD had poor to good reliability for the Cerebral Peduncle approach (ICC > 0.740), good to excellent reliability for the Probabilistic Tract approach (ICC > 0.944), and good to excellent reliability for the Tract Template Approach (ICC > 0.882). ICC values for MD, AD, and RD asymmetries and ratios were similar (Table S1). When individuals with cerebellar or brainstem lesions (*N* = 4) were removed from the FA ICC analysis, the ICC results were similar (difference between ICC with and without these four participants removed was <0.065 across comparisons). Additionally, removing these participants did not change the ratings (i.e., “good” or “excellent”) for any of the FA ICC values.

**Figure 5:**
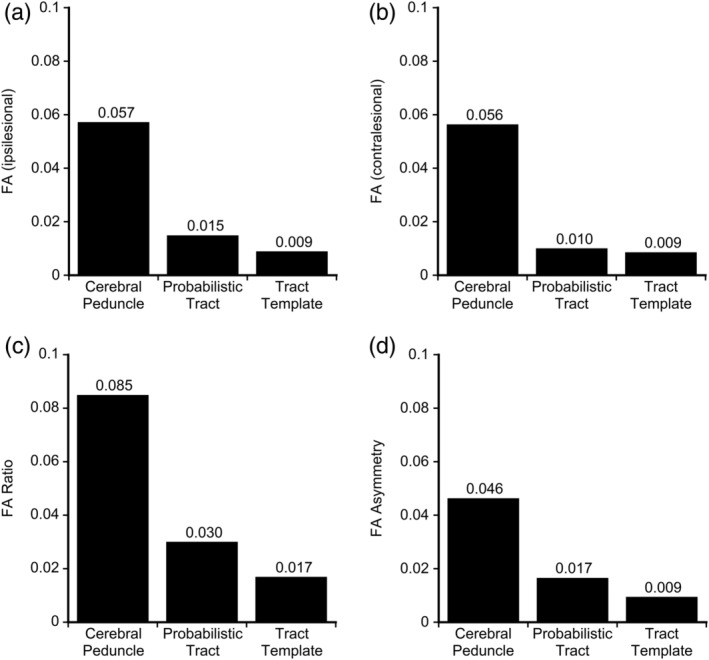
Minimal detectable change (MDC) values of fractional anisotropy (FA) of the corticospinal tract (CST) by approach. MDC values are presented above the individual bars. MDC for FA is presented by approach for (a) Ipsilesional CST, (b) Contralesional CST, (c) FA ratio, and (d) FA asymmetry

MDC_95_ values for FA, MD, AD, and RD are presented in Table 2 and Figure 5. A change in mean FA of 0.057, 0.015, and 0.009 in ipsilesional CST would be needed in order to be 95% confident that this change was greater than the measurement noise for the Cerebral Peduncle, Probabilistic Tract, and Tract template approach, respectively. This pattern, where the Cerebral Peduncle approach requires the largest amount of change to exceed the estimated measurement noise and the Tract Template approach requires the least amount of change to exceed estimated measurement noise, was consistent across all measures of FA, MD, AD, and RD. MDC values for MD, AD, and RD asymmetries and ratios can be found in the Table S1.

## 4 DISCUSSION

All three approaches used in our study showed strong test–retest reliability for evaluating CST integrity. The most automated approach (Tract Template) had the highest reliability followed by the combined manual/automated approach (Probabilistic Tract) then the manual ROI approach (Cerebral Peduncle). FA had higher reliability than MD, AD, or RD. The Tract Template approach had the smallest inherent variability estimated by MDC, followed by the Probabilistic Tract approach, then the Cerebral Peduncle approach. While the Tract Template approach was the most reliable and had the smallest MDC values, FA values evaluated by this approach were not related to behavioral measures of upper extremity impairment. These results have implications for how CST integrity measures may be used in predictive modeling or as a biomarker of the motor system after stroke.

CST integrity is reliable and reproducible in nondisabled adults, including older individuals (Albi et al., 2017; Danielian et al., 2010; Fox et al., 2012; Heiervang et al., 2006; Kristo et al., 2013; Lin et al., 2013; Wakana et al., 2007). Our results showed good to excellent reliability of CST integrity in individuals with chronic stroke, similar to previous studies with smaller sample sizes that used different measurement approaches (Borich et al., 2012; Lin et al., 2013; Snow et al., 2016). In a study of individuals with chronic stroke, fiber assignment by continuous tracking (FACT; ICC = 0.59–0.90) had higher inter‐rater reliability than FA measured by a cross‐sectional manually drawn PLIC ROI (ICC = 0.37–0.71; Borich et al., 2012), suggesting that inter‐rater reliability is higher for more automated approaches than for manual approaches. The results of the current study that utilized different approaches to quantify CST integrity found similar results; the Tract Template and Probabilistic Tract approaches that were more automated had higher test–retest reliability than the Cerebral Peduncle approach which was manual.

While the degree of automaticity may influence reliability, the specific tractography approach (deterministic vs. probabilistic) or diffusion data modeling approach (tensor‐based vs. constrained spherical deconvolution [CSD]) may not. Our DTI‐based Probabilistic Tractography approach produced similar test–retest reliability for CST FA (ICC = 0.93–0.97) to a previous study using deterministic fiber tractography on diffusion data modeled using CSD (ICC = 0.89–1.00; Snow et al., 2016). Differences have been noted in some metrics (number of tracked fibers) between deterministic and probabilistic tractography (Bonilha et al., 2015) and other metrics (mean FA, number of tracts, tract volume) between tensor‐based versus CSD diffusion modeling (Auriat, Borich, Snow, Wadden, & Boyd, 2015). However, the consistency of the ICC values from this study and the previous study by Snow et al. (2016) results suggest that there are not large differences in test–retest reliability between deterministic versus probabilistic tractography or tensor‐based versus CSD diffusion modeling for the CST after stroke. Importantly, this finding may be specific to the reliability of CST measurement (a tract that runs in a uniform direction) and is not a reflection of the validity of tractography approaches (deterministic vs. probabilistic) or diffusion modeling approaches (CSD vs. DTI). Overall, the results of the current study suggest that test–retest reliability of measures of integrity of the CST using a probabilistic tractography approach in individuals with chronic stroke is excellent.

In addition to evaluating FA, MD, AD, and RD, we also examined the reliability of the normalized tract integrity values (FA ratio and FA asymmetry) since these values are commonly related to measures of behavior in chronic stroke (Borich et al., 2012; Cassidy, Tran, Quinlan, & Cramer, 2018; Lindenberg et al., 2010; Stewart, Dewanjee, Shariff, & Cramer, 2016; Stewart et al., 2017; Stinear et al., 2007). FA ratio and FA asymmetry values were reliable across time (ICC = 0.82–0.98), with the mean values reflecting a relatively small difference between ipsilesional and contralesional CST. The small difference between the ipsilesional and contralesional tract could be attributed to our methods; lesioned voxels were not included in data analysis. Removing lesioned voxels from the Tract Template approach provided consistency across all approaches; our participants did not have lesions that overlapped with the cerebral peduncle ROI, and tract drawing algorithms prevent lesioned voxel inclusion. For the Tract Template approach, we masked lesions during the transformation processes using cost functions and utilized an FA threshold to remove lesion voxels from analyses. Removal of lesioned voxels from FA analyses after stroke may impact FA asymmetry and the relationship between FA asymmetry and motor function (Archer, Patten, & Coombes, 2017). Since lesioned voxels generally have a relatively low FA value, ipsilesional FA may have been higher than if lesioned voxels were included, leading to a higher FA ratio and lower FA asymmetry than reported in other studies. Inclusion of lesioned voxels when extracting FA from the CST may provide an FA value that represents lesion load in addition to the integrity of the remaining tract. Distinction between measures of white matter integrity (with lesioned voxels excluded) and lesion load (with lesioned voxels included) may be useful for future research investigating CST integrity as an imaging biomarker of the motor system after stroke.

Our study is the first to report MDC values for CST integrity. MDC reflects the responsiveness of a measure by estimating how much change is required to exceed the inherent noise or variability of the measurement (Portney & Watkins, 2008). In our results, the Cerebral Peduncle approach had the largest MDC, followed by the Probabilistic Tract approach, then the Tract Template Approach. Therefore, according to the current results, to be 95% confident that a change in the ipsilesional CST FA exceeded inherent measurement noise, FA would have to change by 0.045 using the Cerebral Peduncle Approach, whereas a change of 0.009 would exceed measurement noise for the Tract Template Approach. MDC values are important for the interpretation of outcome measurement in clinical intervention trials or longitudinal studies that follow individuals over time. Changes that do not exceed MDC values should not be interpreted as representing real change, even when differences are statistically significant. Recently, studies have begun to examine the effect of training on white matter integrity after stroke (Caeyenberghs et al., 2018; Schlaug et al., 2009; Wan et al., 2014; Zheng & Schlaug, 2015). Changes in DTI‐derived measures of CST integrity should be interpreted within the context of tract and population‐specific MDC values.

While the focus of this study was on reliability and not validity, we did find that the relationship between mean CST FA and level of motor impairment varied between the three approaches. FA from the Cerebral Peduncle approach showed the strongest correlation with motor impairment while FA from the Tract Template approach showed the weakest (and nonsignificant) correlation with motor impairment. FA values vary along the CST with the integrity of inferior, caudal portions of the tract (approximately from the corona radiata to PLIC to CP) often showing the strongest relationships to motor behavior (Archer, Patten, et al., 2017; Schaechter et al., 2009). The ROI used in the Cerebral Peduncle approach captured the caudal portion of CST and may explain why this approach showed the strongest relationship with motor impairment. The lack of a significant correlation between FA and UEFM in the Tract Template approach may be related to noise in the transformation processes. Difficulty with transformation processes in brains with lesions have been documented (Kim et al., 2018), and the optimal procedure for completing transformations in stroke‐lesioned brains remains unknown. Despite utilization of lesion masks in cost functions, our transformations were suboptimal, where the resulting tract mask appeared laterally shifted, not fully capturing the CST (Figure 1c). This poor overlap may have led to the capture of CST‐irrelevant voxels while missing CST‐relevant voxels in data extraction and could have influenced the relatively low correlation between FA and motor behavior. Different transformation approaches that improve the registration process in clinical populations with brain lesions may lead to more valid extraction of CST FA with the Tract Template approach.

The amount of time and expertise required to execute each approach in addition to reliability may need to be considered when selecting a method to evaluate CST integrity. While the Cerebral Peduncle required the least amount of time and expertise, the reliability for this approach was the lowest of the three approaches. The Probabilistic Tract approach showed excellent test–retest reliability and correlated with upper extremity impairment; however, this approach required the most time and expertise to complete. Finally, while the Tract Template approach had excellent test–retest reliability and required minimal to moderate amount of time and expertise, it did not correlate with measures of motor impairment. This tradeoff between level of reliability, correlation with motor impairment, and the amount of expertise/time is relevant for researchers when selecting an approach to measure CST FA in individuals with chronic stroke. Based on the results of the current analysis, we suggest that the Probabilistic Tract approach captures the CST well, showing the expected relationship between CST FA and motor impairment, while offering excellent test–retest reliability and should be used when researcher expertise and time allow.

The range of FA values varied between the three methods. FA values for the ipsilesional CST ranged from 0.432 to 0.673 for the Cerebral Peduncle Approach, 0.403 to 0.521 for the Probabilistic Tract Approach, and 0.387 to 0.468 for the Tract Template Approach (Figure 3). The difference in ranges between the Cerebral Peduncle approach and the two full tract approaches (Probabilistic Tract and Tract Template) may be explained by partial volume effects. Full tract approaches are more likely to include voxels closer to cortical regions that have both white and gray matter resulting in lower, overall mean FA values. Other studies that have used approaches that focus on caudal portions of the CST, either a specific cerebral peduncle ROI or a segment between the PLIC and pons, have found similar larger ranges in FA values (Burke et al., 2014; Feldman, Boyd, Neva, Peters, & Hayward, 2018) while studies that have implemented full tract approaches have found similar tighter ranges with generally lower FA values (Park, Kou, Boudrias, Playford, & Ward, 2013; Snow et al., 2016). The approach selected to extract FA appears to affect the range of mean FA values.

CST integrity has been suggested as a biomarker of the motor system after stroke (Boyd et al., 2017). The reliability, validity, sensitivity, and specificity of measurements are fundamental to utilization of biomarkers (Milot & Cramer, 2008). The results of our study provide valuable information about the stability of DTI‐based measurement of CST integrity over time that may impact the robustness of predictive models and the interpretation of change values in chronic stroke. Previous work has reported on the reliability of CST integrity; however, studies were limited by small sample size, uncontrolled time between test–retest scans, absence of calculations of minimal detectable change, absence of test–retest ICC calculations (only inter‐ and intra‐rater reliability), or by reporting on a single measurement approach. Our results present previously unreported ICCs for test–retest reliability and MDCs for three different approaches with a controlled time between test–retest scans (4 days). Overall, our results found FA to be a reliable measure, supporting its use as a biomarker of the motor system after stroke.

In general, our participants presented with variable lesion size, location, and level of motor impairment. The characteristics of the study population should be considered when interpreting and applying the ICCs and MDCs reported here. Stroke severity may impact the relationship between FA and behavior. While we did not have sufficient power to directly compare a mild/moderate impairment group (*n* = 13) to a severe impairment group (*n* = 5), previous studies suggest brain–behavior relationships differ based on motor severity (Feldman et al., 2018; Quinlan et al., 2018; Stewart et al., 2017). In addition, our participants were all in the chronic phase of stroke; the reliability of measures of CST integrity may be different in individuals in the acute or subacute phase of stroke recovery. We took precautions to limit experimenter bias by blinding the researchers performing data analysis to the participant's identification and day of data collection. However, we cannot rule out potential bias from possible identification of the lesioned hemisphere during mask drawing, which would have been especially apparent in individuals with large lesions. In addition, the period of practice of a joystick task between imaging collection may have impacted our results. While the amount of practice between scans was below the expected dosage to impact brain structure, the practice could have resulted in larger differences between test and retest measures, as well as lower reliability, if it did impact the structural integrity of the CST. Finally, all data was extracted from each participants’ native space. Therefore, researchers were not able to standardize the location of the ROI masks using MNI coordinates. While this likely introduced variability in the spatial location of masks, extracting diffusion data from native space was performed to limit the effect of the transformation process on data values.

## 5 CONCLUSION

DTI‐based measures of CST integrity showed good to excellent test–retest reliability in individuals in the chronic phase of stroke. More automated approaches (Probabilistic Tract and Tract Template approach) were more reliable than manual approaches (Cerebral Peduncle approach). However, more research is needed to determine other important psychometric properties of white matter integrity measurements like sensitivity, specificity, and validity, especially considering the variable relationship between CST FA and motor impairment depending on the measurement approach. There is a tradeoff when selecting an approach for measuring CST integrity, where no single approach appears to be superior in reliability, correlation with motor impairment, amount of expertise required, and amount of time required. Examination of the psychometric properties of diffusion tensor measurements in larger populations will be important for identifying optimal processes for quantifying white matter integrity.

## CONFLICT OF INTEREST

The authors have no known conflicts of interest to disclose.

## Supporting information


**Appendix S1** Supporting Information.

## Data Availability

The data used in this study are not publicly available but are stored by the principal investigator and are available from the corresponding author upon reasonable request.
