# Supplementary material for: Test–retest reliability and minimal detectable change of corticospinal tract integrity in chronic stroke
Source: Hum Brain Mapp. 2020 Feb 24;41(9):2514–26. doi: 10.1002/hbm.24961 (PMC7268047; doi:10.1002/hbm.24961)
Supplement: Supplementary file 1 — Appendix S1 Supporting Information. [file HBM-41-2514-s001.docx]

**Supplemental Data**

**Test-retest reliability and minimal detectable change of corticospinal tract integrity in chronic stroke**

Allison Foster Lewis^a^, Makenzie Myers^a^, Jenny Heiser^a^, Melissa Kolar^a^, Jessica F. Baird^a^, Jill Campbell Stewart^a*^

^a^Department of Exercise Science, University of South Carolina, Columbia, SC 29208, USA

*Corresponding author at: Jill Campbell Stewart

University of South Carolina

921 Assembly Street, Room 301D

Columbia, SC 29201, USA

Email address: jcstewar@mailbox.sc.edu

| **Supplemental Tables and Figure**  Supplemental Table | | | | | | | |
| --- | --- | --- | --- | --- | --- | --- | --- |
|  | | Mean Day 1 | Mean Day 4 | ICC | ICC CI | ICC Rating | MDC_95_ |
| **Cerebral Peduncle** | | | | | | | |
|  | *MD (x10^-3^ mm^2^/s)* |  |  |  |  |  |  |
|  | Ratio | 1.119  (0.126) | 1.111  (0.103) | 0.624^†^ | 0.227-0.841 | Moderate | 0.214 |
|  | Asymmetry | -0.053  (0.056) | -0.051  (0.046) | 0.627^†^ | 0.230-0.843 | Moderate | 0.094 |
|  | *AD (x10^-3^ mm^2^/s)* |  |  |  |  |  |  |
|  | Ratio | 1.054  (0.105) | 1.046  (0.099) | 0.806^††^ | 0.554-0932 | Good | 0.129 |
|  | Asymmetry | -0.024  (0.050) | -0.021  (0.046) | 0.800^††^ | 0.542-0.920 | Good | 0.062 |
|  | *RD (x10^-3^ mm^2^/s)* |  |  |  |  |  |  |
|  | Ratio | 1.220  (0.194) | 1.211  (0.150) | 0.558^†^ | 0.125-0.810 | Moderate | 0.357 |
|  | Asymmetry | -0.093  (0.077) | -0.091  (0.062) | 0.578^†^ | 0.153-0.820 | Moderate | 0.139 |
|  |  |  |  |  |  |  |  |
| **Probabilistic Tract** | |  |  |  |  |  |  |
|  | *MD (x10^-3^ mm^2^/s)* |  |  |  |  |  |  |
|  | Ratio | 1.099  (0.108) | 1.102  (.0109) | 0.994^††^ | 0.984-0.998 | Excellent | 0.023 |
|  | Asymmetry | -0.045  (0.046) | -0.046  (0.047) | 0.993^††^ | 0.982-0.997 | Excellent | 0.011 |
|  | *AD (x10^-3^ mm^2^/s)* |  |  |  |  |  |  |
|  | Ratio | 1.063  (0.093) | 1.065  (0.094) | 0.992^††^ | 0.980-0.997 | Excellent | 0.023 |
|  | Asymmetry | -0.029  (0.042) | -0.030  (0.042) | 0.991^††^ | 0.977-0997 | Excellent | 0.011 |
|  | *RD (x10^-3^ mm^2^/s)* |  |  |  |  |  |  |
|  | Ratio | 1.141  (0.129) | 1.145  (0.130) | 0.991^††^ | 0.976-0.996 | Excellent | 0.034 |
|  | Asymmetry | -0.063  (0.054) | -0.065  (0.054) | 0.990^††^ | 0.975-0.996 | Excellent | 0.015 |
|  |  |  |  |  |  |  |  |
| **Tract Template** | | | | | | | |
|  | *MD (x10^-3^ mm^2^/s)* |  |  |  |  |  |  |
|  | Ratio | 1.095  (0.105) | 1.091  (0.103) | 0.992^††^ | 0.980-0.997 | Excellent | 0.026 |
|  | Asymmetry | -0.043  (0.046) | -0.041  (0.045) | 0.991^††^ | 0.977-0.997 | Excellent | 0.012 |
|  | *AD (x10^-3^ mm^2^/s)* |  |  |  |  |  |  |
|  | Ratio | 1.057  (0.081) | 1.055  (0.082) | 0.933^††^ | 0.981-0.997 | Excellent | 0.058 |
|  | Asymmetry | -0.026  (0.040) | -0.025  (0.037) | 0.992^††^ | 0.979-0.997 | Excellent | 0.009 |
|  | *RD (x10^-3^ mm^2^/s)* |  |  |  |  |  |  |
|  | Ratio | 1.135  (0.131) | 1.128  (0.128) | 0.991^††^ | 0.974-0.997 | Excellent | 0.035 |
|  | Asymmetry | -0.060  (0.055) | -0.057  (0.054) | 0.989^††^ | 0.971-0.996 | Excellent | 0.016 |

Mean values (standard deviation); FA = fractional anisotropy; MD = mean diffusivity (×10^-3^ mm^2^/s); AD = axial diffusivity (×10^-3^ mm^2^/s); RD = radial diffusivity (×10^-3^ mm^2^/s); FA ratio = FA_ipsilesion_/FA_contralesion_; FA asymmetry = (FA_contralesion_ -FA_ipsilesion_)/(FA_contralesion_ + FA_ipsilesion_); ICC = intraclass correlation coefficient; CI = 95% confidence interval; MDC_95_ = minimal detectable change at the 95% confidence interval; * = significant difference at p<0.05 between ipsilesional value and contralesional value; ^††^ = F-statistic for the ICC is significant at p<0.001; ^†^ = F-statistic for the ICC is significant at p<0.02

Supplemental Figure


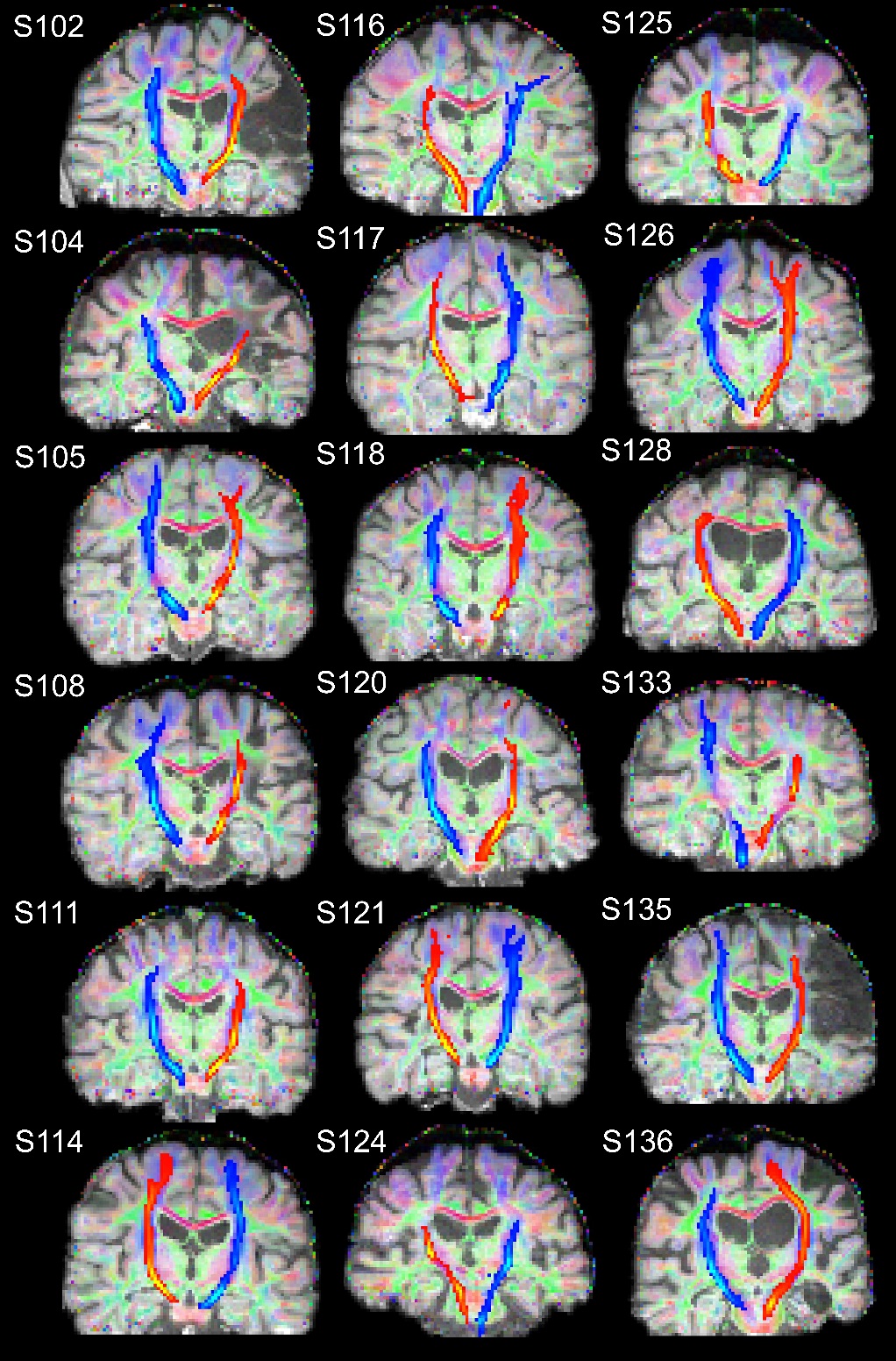


Supplemental Figure. Individual subject results from probabilistic tractography seeded from M1 in a single coronal slice. The red-yellow indicates the ipsilesional CST and the blue-turquoise indicates the contralesional CST. Continuous tracts were obtained for all participants.
